# Coffee Biotransformation in Volcanic Process: A Chemical and Sensory Analysis

**DOI:** 10.3390/foods14081368

**Published:** 2025-04-16

**Authors:** Renata A. R. Rocha, Lívia C. F. Silva, Marcelo A. D. da Cruz, Luiza M. A. B. Cardoso, Arlley de B. M. Sousa, Laila Alonso, Marcela V. C. Machado, Gisele X. R. Costa, Laurence R. Amaral, Pedro L. L. Bertarini, Matheus S. Gomes, Líbia D. Santos

**Affiliations:** 1 Biotechnology Institute, Federal University of Uberlândia, Patos de Minas 38702-178, MG, Brazil; renata.reis@ufu.br (R.A.R.R.); livia.fidelis@ufu.br (L.C.F.S.); marcelo.antonio@ufu.br (M.A.D.d.C.); 2 Faculty of Chemical Engineering, Federal University of Uberlândia, Patos de Minas 38702-178, MG, Brazil; luiza.cardoso@ufu.br (L.M.A.B.C.); arlleybmsousa@ufu.br (A.d.B.M.S.); laila.alonso@ufu.br (L.A.); marcela.machado@ufu.br (M.V.C.M.); gisele.ribeiro@ufu.br (G.X.R.C.); 3 Laboratory of Bioinformatics and Molecular Analysis (LBAM), Federal University of Uberlândia, Patos de Minas 38702-178, MG, Brazil; laurence@ufu.br (L.R.A.); matheusgomes@ufu.br (M.S.G.); 4 Faculty of Electrical Engineering, Federal University of Uberlândia, Patos de Minas 38702-178, MG, Brazil; bertarini@ufu.br

**Keywords:** volcano method, coffee quality, fermentation techniques, specialty coffee, post-harvest processing

## Abstract

Volcanic fermentation is an innovative technique in post-harvest coffee processing that involves forming conical mounds, called “volcanoes”, to create specific biotransformation conditions. This study investigates the impact of different volcano fermentation methods on the chemical composition and sensory attributes of coffee. Four methods were evaluated: asphalt patio (E1), on pallets (E2), in steel containers under the sun (E3), and in steel containers in the shade (E4). The chemical composition was analyzed in terms of sugars (sucrose, glucose, fructose), organic acids (citric, malic, succinic, lactic, acetic) and alcohols (glycerol, ethanol). In addition, color differences (ΔE) and sensory analysis of the fermented coffees were evaluated. The results of this study reveal that volcanic fermentation produces high-quality specialty coffees, but with divergent profiles of acids and alcohols, thus influencing the sensory characteristics of the resulting beverage. However, the different methods of volcanic fermentation did not significantly affect pH and soluble solids, indicating that the microbiota developed an efficient and consistent fermentation regardless of the solar exposure conditions. The most frequently mentioned sensory descriptors were chocolate, citrus fruits, honey/molasses, caramel, floral, and brown sugar. These findings highlight the significant influence of the volcanic fermentation method on the chemical and sensory quality of coffee fermented.

## 1. Introduction

During the post-harvest processing of coffee, fermentation occurs spontaneously by microorganisms such as bacteria, yeasts, and fungi from the natural microbiota present in the fruits. These microorganisms produce different enzymes and metabolites using various compounds to survive [1]. Polysaccharides are hydrolyzed into simple sugars and are used to produce alcohol, mannitol, glycerol, and acids such as citric, malic, succinic, lactic, oxalic, acetic, isobutyric, and propionic acids [2,3,4,5]. Proteins are hydrolyzed into amino acids and metabolized into alcohols, aldehydes, and sulfur compounds; lipids into fatty acids that form esters, methyl ketones, alcohols, and lactones; and chlorogenic acids into phenolic acids [3,5,6].

The coffee fermentation process is a complex and multivariate system influenced by various environmental conditions, such as temperature, pH, microbiota, and the presence of water and oxygen [7,8,9]. Different fermentation methods can lead to significant variations in chemical composition and sensory attributes, and choosing the right method can optimize the quality of the final product. Depending on the type of environmental conditions, fermentation can occur in an open or closed environment [3].

The volcanic fermentation method is a biotechnological process that occurs in open environments, commonly used in Cerrado Mineiro, Brazil. Initially employed as an alternative drying method on patios to save space, it was observed that slower drying enhanced the sensory quality of the coffee beverages. Thus, its potential as a fermentative method was later recognized. This process involves creating “volcanoes” of coffee cherries, which are piles of coffee arranged in a conical shape, creating anaerobic microenvironmental conditions that favor microbial interactions and physical conditions that enhance the fermentation process. Volcanic biotransformation can offer more effective control of temperature and humidity, intensify the coffee’s sensory profile, and promote a microbial diversity that results in a more uniform, rich, and varied fermentation, allowing for the exploration of a more complex and pleasant sensory profile.

Fermentation, as a critical post-harvest process, can be manipulated to improve coffee quality through microbial activity that generates essential metabolites, forming distinct sensory profiles and enhancing the final quality of the beans [10,11,12,13]. This improvement in sensory quality is attributed to changes in the chemical composition of the coffee bean, significantly influenced by factors such as microorganisms, enzymatic reactions, and environmental parameters, including temperature, the presence or absence of oxygen, and the duration of the process [14]. Thus, distinct flavors and high complexity are used as parameters in the characterization of specialty coffees. [15].

Recent studies have highlighted the crucial role of specific microorganisms in the formation of flavor-active metabolites during coffee fermentation. *Saccharomyces cerevisiae*, one of the most commonly found yeasts in spontaneous fermentations, contributes significantly to the production of ethanol and glycerol, which enhance body and sweetness perception in coffee beverages [1,16]. *Lactobacillus plantarum*, a prevalent lactic acid bacterium, is associated with the biosynthesis of lactic and acetic acids, influencing acidity and complexity [4,17]. Moreover, non-Saccharomyces yeasts such as *Pichia kudriavzevii* have demonstrated the ability to produce fruity esters and higher alcohols that contribute to floral and tropical fruit notes [6]. These microbial species interact synergistically, and their metabolic activity is modulated by environmental factors such as temperature, oxygen availability, and substrate composition, ultimately shaping the sensory attributes of the final product [10,18]. Understanding the specific microbial contributions is essential for optimizing fermentation protocols aimed at enhancing specialty coffee quality.

Although promising, the volcanic biotransformation of coffee still presents considerable challenges, particularly regarding the control of fermentation conditions and the complex dynamics of microbial interactions. To date, scientific investigations exploring this technique remain scarce, highlighting the need for studies aimed at elucidating the environmental and microbial factors involved in the process, as well as strategies for its optimization. In this context, the present study evaluates the impact of different volcanic biotransformation approaches on the chemical composition and sensory profile of Arabica coffee cv. Paraíso, using the native microbiota naturally present in each experimental condition.

## 2. Materials and Methods

### 2.1. Fermentation Process

The Coffea arabica cv Paraíso, cultivated at an altitude of 1050 m at Fazenda Kaizen, located in Patos de Minas, in the Cerrado Mineiro region, State of Minas Gerais, Brazil, was fermented using the volcanic process. The processing of the coffee cherries was carried out on the same day as the harvest, which included washing with water to remove impurities and classification according to the stage of ripeness.

In the fermentative process, 4000L of coffee cherries was arranged in a volcano shape. The ‘volcano’ configuration consisted of a conical pile of coffee fruits manually arranged to simulate the geometry of a volcanic slope. This shape was designed to enhance internal heat retention and airflow through natural convection, promoting the establishment of thermal and microbial gradients during fermentation. No physical structure was used to contain the pile; the shape was maintained by manual stacking. The experiment was conducted under open environmental conditions for 36 h.

**Figure 1:**
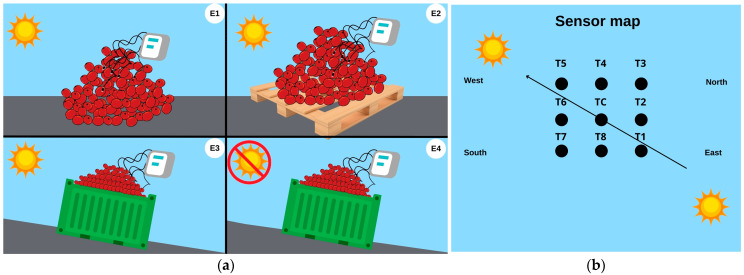
Experimental design: (**a**) experimental setup illustrating the volcanic biotransformation process; (**b**) location map of temperature sensors. E1: asphalt patio, E2: on pallets E3: in steel containers under the sun, E4: in steel containers in the shade. T1 was located on the east side (sunrise), and T5 on the west side (sunset). Sensors T2 and T4 were placed at central and northern positions, respectively, while T3 was on the northeast side. T6, T7, and T8 were positioned toward the southern and central-western areas. TC represents the geometric center of the volcano. Note: the containers used in experiments E3 and E4 were inclined at 45° to simulate the slope of a volcanic surface and to promote internal heat circulation.

To monitor the temperature at different points of the volcanoes, electronic devices called “dataloggers” were used throughout the 36 h of fermentation. The temperature sensors were arranged according to Figure 1. It is worth noting that T1 was positioned to the east (sunrise) and T5 to the west (sunset) in all experiments. The experiment was conducted in June, which had approximately 11 h and 14 min of sunlight exposure.

In the experiments with the direct volcano on the patio (E1) and the volcano isolated by pallet (E2), biotransformations occurred under sunlight exposure. Both E1 and E2 were conducted on a flat patio surface; however, in E2, the coffee cherries were separated from the ground by a wooden pallet. In the other treatments—volcano sun (E3) and volcano shade (E4)—the coffee cherries were placed in metal containers inclined at a 45° angle to allow for the drainage of fermentation exudates, under sunlight and shaded conditions, respectively (Appendix A). Photographs of the experimental setups and coffee samples are provided in the Supplementary Materials (Figure S1). In E3 and E4, the inclination of the containers enhanced internal heat distribution and mimicked a volcanic slope configuration.

Temperature sensors (T1 to T8) were positioned at the bottom of the coffee mass, distributed across the east, west, center, north, and south regions to monitor internal heat gradients. Surface temperatures were measured manually with a digital thermometer at the same locations as the sensors at each sampling time.

After fermentation, the coffee cherries were washed with water and then subjected to drying on raised beds. This drying process continued until the beans reached a moisture content of approximately 12%. After drying, the beans were stored and left to rest for 30 days before proceeding with the coffee-processing stages.

### 2.2. Physicochemical Analyses

#### 2.2.1. Soluble Solids, pH and Temperature

Ten repetitions of each analysis were performed both at the beginning (0 h) and at the end of fermentation (36 h). The soluble solid content, measured in degrees Brix, was determined using a digital optical refractometer, model MA888, from Akso^®^. Temperature and pH measurements were conducted using a pH meter for semi-solids, also from Akso^®^. These analyses were performed according to the methods recommended by the AOAC [19].

#### 2.2.2. Texture Profile (TPA)

To evaluate the texture profile (TPA) of green coffee beans, a TA-XT2i Stable Micro System texture analyzer was used, focusing on determining hardness parameters. A cylindrical aluminum probe, 25 mm in diameter, TPA macro—AIBCAKE 2, was used for this purpose. The probe was positioned 40 mm above the sample, with a return speed of 30 mm/second and a contact force of 30 g. Data collection was performed using the software ‘Texture Expert for Windows’, version 1.20 (Stable Micro Systems, Godalming, UK). Five measurements per sample were taken to assess the impact of the process on the bean’s resistance to breakage.

#### 2.2.3. Color Analysis

Color measurements were conducted using a DeltaVista^®^ colorimeter, which provided readings for the L (lightness), a (red-green), and b* (yellow-blue) coordinates on processed green coffee bean samples. Each sample was analyzed in ten replicates. The total color difference (ΔE) between the control and fermented samples was calculated according to Equation (1):

ΔE = √[(ΔL*)^2^ + (Δa*)^2^ + (Δb*)^2^] (1)

ΔE: total color difference between control and fermented beans;
ΔL: Difference in lightness (L*) between control and fermented beans;
Δa: difference in the red–green axis (a*) between control and fermented beans;
Δb: difference in the yellow–blue axis (b*) between control and fermented beans.

### 2.3. High-Performance Liquid Chromatography (HPLC) Metabolites Analysis

Using High-Performance Liquid Chromatography (HPLC), the consumption of sugars (sucrose, fructose, and glucose) and the production of organic acids (malic, lactic, acetic, butyric, propionic, citric, and succinic) and alcohols (ethanol and glycerol) were evaluated according to the methodology adapted from ELHALIS et al. [6]. For this, in the preparation of the samples, fruits collected at the beginning and end of the fermentations, 10 g of homogenized fruits with 100 mL of deionized water was added to a domestic blender (Oster 1400 W) for 2 min. The mixture obtained was filtered through four layers of polypropylene fabric and then centrifuged for 15 min at 17 °C in a Heal Force Neofuge 18R centrifuge at 13,000 rpm. The supernatant was collected and filtered through a 0.22-micrometer nylon filter before being added to the Shimadzu HPLC system for analysis (Shimadzu Corp., Kyoto, Japan). The prepared samples were examined by a Shimadzu HPLC system, model LC-20A Prominence, equipped with a SUPELCOGEL C-610H column (30 mm × 7.8 mm). For mobile phase preparation, a 0.1% aqueous phosphoric acid solution was used, operating with an eluent flow rate of 0.5 mL/min. For this analysis, the column temperature was 32 °C, and the analyses lasted 35 min with a volume of 20.0 μL. Sugar and ethanol concentrations were analyzed using a refractive index detector, while acids were identified using a photodiode array detector (PDA) at 210 nm. The obtained data were processed with LC-Solutions software (version 5.117), using specific calibration curves for each compound.

### 2.4. Sensory Analysis Methodology

This study was reviewed and approved by the Ethics Committee of the Federal University of Uberlândia (CAAE: 73864723.0.0000.5152). To perform the sensory analysis, coffee beans were selected and roasted at the Expocacer roasting facility, located in Patrocínio, Minas Gerais, Brazil, according to the guidelines of the Specialty Coffee Association (SCA). The medium roast was conducted no more than 24 h before tasting, with a roasting duration ranging between 8 and 12 min. Before the sensory analysis, the beans were ground using an electric grinder (Tramontina, Rio Grande do Sul, Brazil). For the sensory evaluation of the coffee beverage, the roasted beans were prepared according to the SCA protocol. Five cups were prepared for each sample, maintaining a precise ratio of 8.25 ± 0.25 g of coffee to 150 mL of water, with the water temperature controlled between 92.2 °C and 94.4 °C.

Five certified Q-Graders evaluated the sensory attributes of the coffee samples using Cropster^®^ software (web version). They analyzed 10 specific attributes: fragrance/aroma, flavor, aftertaste, acidity, body, balance, uniformity, clean cup, sweetness, and overall quality, in addition to identifying defects. Each attribute was scored on a scale from 6 to 10, with increments of 0.25. Negative attributes indicating bad or undesirable flavors were classified as defects and negatively impacted the overall quality index of the coffee. Besides these quantitative evaluations, the tasters also assigned descriptive sensory attributes to each sample, using the descriptors provided by the Flavor Wheel [20].

To evaluate the description of the sensory descriptors, stacked column charts were used. In this method, each column is divided into segments that correspond to the different sensory descriptors, identified by specific colors. The segments are expressed as percentages, reflecting the frequency of citations made by the five evaluators (Q-Graders).

### 2.5. Data Processing and Multivariate Analysis

For the analysis of the physicochemical and sensory data, Shapiro–Wilk normality tests were conducted to verify whether they followed a normal distribution. For those with a normal distribution, the comparison between the means of the treatments was performed using the Sensomaker^®^ software (version 1.94). The means were compared using ANOVA, followed by Tukey’s post hoc test for significant differences (*p*-value ≤ 0.05).

With the data from the coffee sensory analyses, the decision tree classification method was used, a machine learning technique that identifies the key attributes for the accurate classification of subgroups within this study. To test and validate the accuracy of the decision trees, the ‘full-training-cross-validation’ method was applied. This method involves using all samples from the dataset for both training and testing, resulting in the percentage of errors identified, which offers a robust assessment of the model’s effectiveness (Matlab version 2023). Additionally, the temperature datasets were analyzed using heatmap analysis for a visual representation of the data patterns.

## 3. Results

### 3.1. Temperature

**Figure 2:**
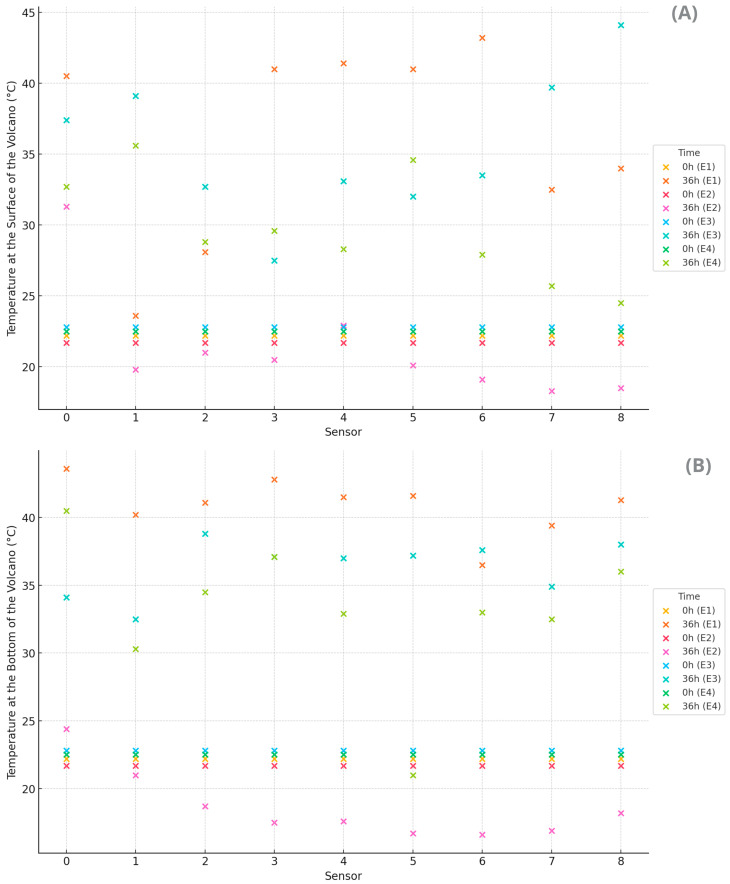
Variation in final temperature in the fermentation process. E1: asphalt patio, E2: on pallets E3: in steel containers under the sun, E4: in steel containers in the shade. Data are shown as the mean. (**A**,**B**) refers to temperature at the surface and bottom of the volcano, respectively.

The coffee masses generated enough heat to alter the thermodynamic equilibrium with the environment during fermentation in all volcanic processes (Figure 2). The volcano method, conducted on the asphalt patio, showed elevated temperatures both on the surface (Figure 2A) and at the bottom (Figure 2B). The bottom center was warmer, while the surface exhibited greater thermal variation. The higher overall temperatures indicate good heat retention, especially in the center, which is beneficial for more efficient fermentation by accelerating the desired biochemical reactions. However, there is a risk of overheating in some areas. It is also worth noting that solar exposure influenced the observed temperatures, with lower readings to the east (sunrise) and higher readings to the west (sunset).

On the other hand, the volcano method on a pallet exhibited uniform distribution but with low temperatures at both levels, suggesting a more controlled fermentation due to the insulation provided by the pallet. The ventilation provided by the pallet resulted in lower temperature increases, which may indicate lower fermentative activity or better heat dissipation. The smaller temperature variation compared to the bottom suggests that the pallet insulated and hindered heat transfer by conduction from the asphalt to the mass of coffee cherries. The use of the pallet indicated a delay in biochemical reactions and affected the development of flavor compounds.

The volcano method in an open container under sun exposure showed high heat retention, especially on the surface, accelerating fermentation but with the risk of uneven fermentation and hotspots (T8). Additionally, it had a hotter surface and moderate bottom, reflecting the interference of solar exposure.

In the volcano method in an open container conducted in the shade, moderate temperatures were observed, especially on the surface, suggesting slower and more uniform fermentation, ideal for more precise process control. Thus, the bottom was warmer compared to the surface, reflecting that the shade reduced the thermal interference of solar radiation on the surface. The shade affected heat retention, resulting in lower temperatures at the edges. However, variations are still notable due to the steel structure. Although there is heat retention in the center, the cold edges can create inconsistent fermentation conditions, impacting the process’s heterogeneity. The surface temperature was more uniform compared to the other experiments.

The container methods (E3 and E4) showed intermediate temperatures, with E3 (under the sun) showing better heat retention compared to E4 (in the shade). Additionally, it showed mixed heat retention, with a large variation between the center and the edges. In all methods, the central treatments tended to show higher temperatures both on the surface and at the bottom, suggesting better heat retention in the volcano’s center, while the treatments at the edges showed lower temperatures, indicating greater heat loss to the environment. This was expected due to the surrounding mass of beans acting as insulators, providing the center coffee beans with less contact with the environment.

The different volcano methods and experimental treatment locations showed significant variations in the bottom and surface temperatures of the volcanoes, directly influencing the efficiency and thermal uniformity of cherry coffee fermentation. Methods that allow for better heat dissipation, such as the volcano on a pallet, showed more controlled temperatures, while methods that retain more heat, such as the volcano container under the sun, can accelerate fermentation but with the risk of overheating. It is important to consider that solar exposure can significantly influence the process due to sunrise and sunset exposure, shade, and other environmental factors.

### 3.2. Colorimetry and Texture Analysis

**Figure 3:**
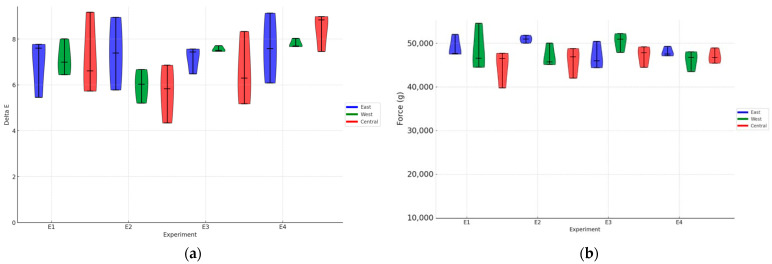
Instrumental analysis of green coffee beans. (**a**) Total color variation (ΔE) between control and fermented samples; (**b**) compression force (N) indicating bean texture. E1: asphalt patio, E2: on pallets E3: in steel containers under the sun, E4: in steel containers in the shade. Data are shown as the mean. ± = standard deviation.

The color analysis results did not show a significant difference (*p* > 0.05) between the experiments or between the positions regarding solar exposure. However, all experimental results showed ΔE values between 5.5 and 8.5 (Figure 3a). Currently, there is no national or international standard that establishes these limits of acceptability. These results indicate that the intensity and uniformity of fermentation vary according to the specific conditions of each treatment, influencing the formation of color compounds in green coffee. However, since the analysis was performed on green coffee, this difference may be minimized with the coffee-roasting process.

Additionally, it was observed that the fermentation process did not significantly alter the strength parameters of the beans (Figure 3b). The textural characteristics of the coffee beans, such as strength, remained relatively unchanged regardless of the post-processing treatment, indicating that these aspects of bean quality are not affected by volcanic fermentation conditions.

### 3.3. pH and Brix

**Figure 4:**
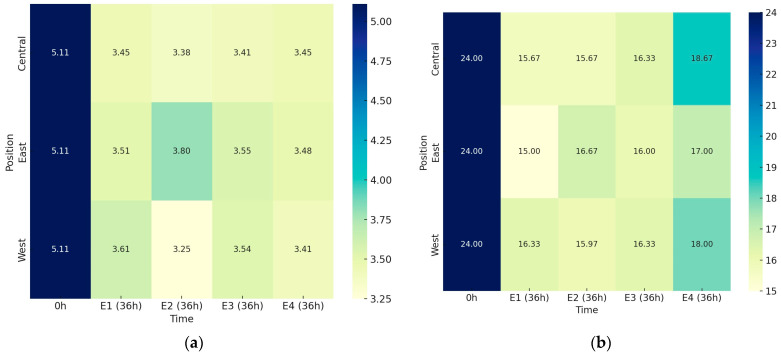
Heatmap showing the variation in (**a**) pH and (**b**) soluble solid concentration (°Brix) during the coffee fermentation process. E1: asphalt patio, E2: on pallets E3: in steel containers under the sun, E4: in steel containers in the shade. Data are shown as the mean.

The metabolic activity of microorganisms during the fermentation of coffee fruit caused a significant decrease in pH values, from 5.11 at the start (0 h) to between 3.80 and 3.25 after 36 h (36 h). This decrease was evident across all four experiments when analyzing the average values of sensors positioned to the east, west, and center (Figure 4a). However, no statistical differences in pH were observed between the four experiments after 36 h (*p* > 0.05) or in the sample positions relative to the sun.

The pH data, along with the treatment positions and volcano configurations, indicate that fermentation is strongly influenced by heat distribution. Methods that retain more heat, such as the volcano on the asphalt patio and the container under the sun, showed more intense fermentations, reflected in greater pH drops. Conversely, methods that allow for better heat dissipation, such as the pallet, resulted in more heterogeneous fermentations.

The concentration of soluble solids significantly reduced in all experiments compared to the initial values (0 h), as shown in Figure 4b. Methods that retain more heat, such as the volcano on the asphalt patio (E1) and the container under the sun (E3), did not show greater reductions in Brix values. Neither did the shade delay fermentative reactions. Different positions related to sun exposure also did not influence Brix variations. The microbiota appeared unaffected by the different conditions, with fermentation occurring continuously and homogeneously.

The concentration of soluble solids for E1 showed a significant reduction in all treatments compared to the initial values (0 h), with variations ranging from 15.00 °Brix (East) to 16.33 °Brix (West). For E2, the final Brix values varied from 15.67 (Central) to 16.67 (East). The variation in soluble solid content for E3 ranged from 16.00 (East) to 16.33 (West and Central). For E4, the final Brix values ranged from 17.00 (East) to 18.67 (Central). All fermentations showed similar behavior concerning the reduction in Brix values.

### 3.4. Sugar Consumption and Production of Organic Acids and Alcohols

**Figure 5:**
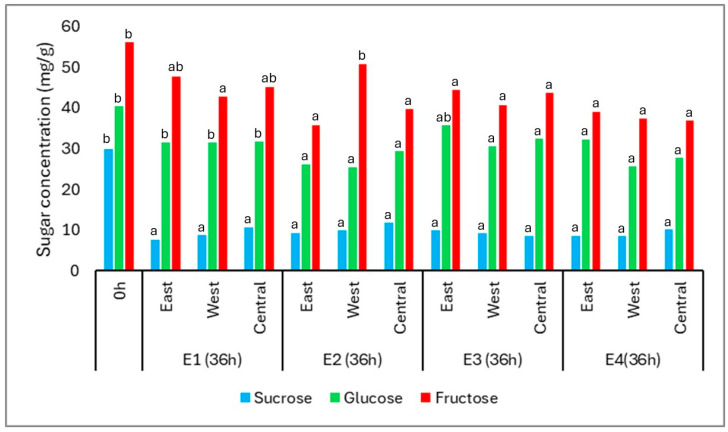
Concentration of sugars (mg/g) in coffee samples before fermentation (0 h) and after 36 h for each position (East, West, and Central) across four treatments: E1 (asphalt patio), E2 (on pallets), E3 (in steel containers under the sun), and E4 (in steel containers in the shade). The sugars evaluated include sucrose, glucose, and fructose. The results represent mean values. Different lowercase letters indicate statistically significant differences between the samples (Tukey’s test, *p* ≤ 0.05). The *Y*-axis represents sugar concentration in mg per gram of sample.

At the beginning of fermentation (0 h), the coffee cherries contained three major sugars: fructose (56.26 mg/g), glucose (40.41 mg/g), and sucrose (29.84 mg/g). After 36 h, all sugars exhibited a general decrease in concentration, reflecting microbial consumption during the fermentation process (Figure 5).

Fructose remained the most abundant sugar throughout, although it showed significant reductions in most treatments and positions, except in E1-E, E1-C, and E2-W. Glucose concentrations decreased significantly in all conditions, especially in E2 and E4, where the final averages reached 27.02 mg/g and 28.49 mg/g, respectively. This behavior is consistent with the higher affinity of yeasts and lactic acid bacteria for glucose as a primary energy source.

Sucrose showed the most pronounced decline, dropping from 29.84 mg/g to an average of 9.36 mg/g across treatments. This likely results from hydrolysis via invertase activity, followed by rapid microbial assimilation of the resulting monosaccharides. The progressive reduction in all three sugars confirms active microbial metabolism during the fermentation period.

**Figure 6:**
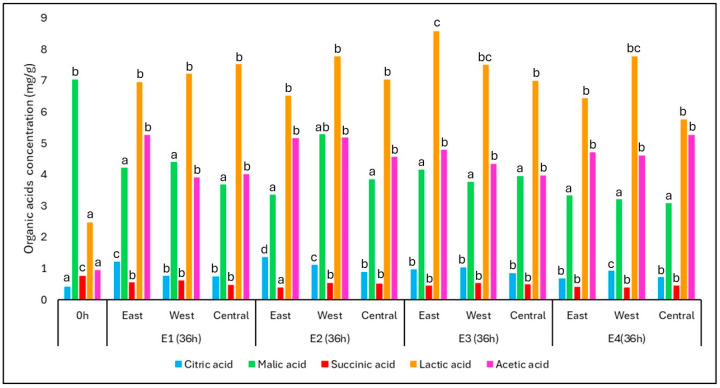
Concentration of organic acids (mg/g) in coffee samples before fermentation (0 h) and after 36 h for each position (East, West, and Central) across four treatments: E1 (asphalt patio), E2 (on pallets), E3 (in steel containers under the sun), and E4 (in steel containers in the shade). The acids evaluated include citric, malic, succinic, lactic, and acetic. The results represent mean values. Different lowercase letters above the bars indicate statistically significant differences (Tukey’s test, *p* ≤ 0.05). The *Y*-axis represents organic acid concentration in mg per gram of sample.

During fermentation, organic acids played a key role as both metabolic intermediates and microbial byproducts. The changes in the concentration of key organic acids throughout the fermentation process are presented in Figure 6, which illustrates their levels before (0 h) and after 36 h of fermentation for each position (East, West, and Central) and treatment (E1 to E4). Among them, lactic and acetic acids showed the most pronounced increases, particularly under the thermally favorable conditions of E3 and E4. These increases are indicative of the activity of lactic acid bacteria (LAB) and acetic acid bacteria (AAB), which thrive in microaerobic and warm environments.

The concentration of lactic acid rose significantly across all treatments, reaching up to 8.58 mg/g in E3-East after 36 h, compared to the initial concentration of 2.48 mg/g. Similarly, acetic acid increased from 0.95 mg/g at time zero to final values averaging 4.65 mg/g, with all treatments presenting consistent accumulation. These trends align with the expected metabolism of *Lactobacillus* and *Acetobacter* spp., frequently reported in spontaneous coffee fermentations.

In contrast, malic acid, which was initially present at 7.03 mg/g, decreased significantly to an average of 3.86 mg/g, reflecting its degradation by both yeasts and LAB, particularly via malolactic and malo-ethanolic pathways. Succinic acid, which had the highest initial concentration among the minor acids (0.77 mg/g), also declined over time, suggesting it was either metabolized or diluted by microbial activity.

Interestingly, citric acid, which began at a low concentration (0.42 mg/g), increased significantly in all treatments, especially in the east positions of E1 and E2. This may be related to microbial synthesis under specific fermentation conditions, though citric acid can also be stabilized or concentrated due to fruit dehydration in warmer zones.

**Figure 7:**
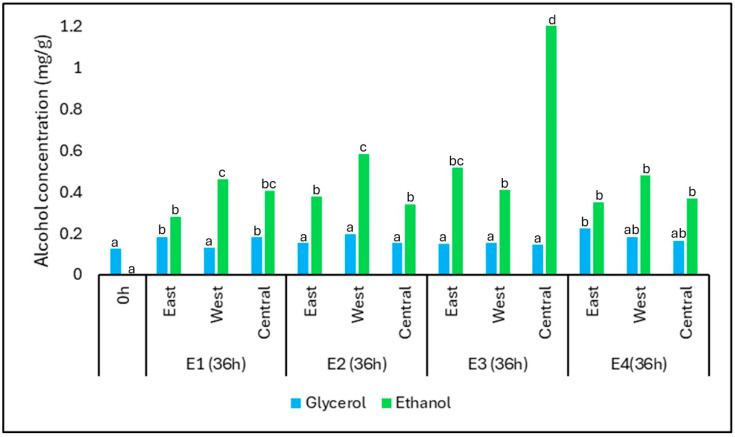
Concentration of alcohols (mg/g) in coffee samples before fermentation (0 h) and after 36 h for each position (East, West, and Central) across four treatments: E1 (asphalt patio), E2 (on pallets), E3 (in steel containers under direct sunlight), and E4 (in steel containers in the shade). Alcohols evaluated include ethanol and glycerol. The results represent mean values. Different lowercase letters above the bars indicate statistically significant differences (Tukey’s test, *p* ≤ 0.05). The *Y*-axis represents alcohol concentration in mg per gram of sample.

The concentrations of ethanol and glycerol before and after fermentation under different treatments and positions are shown in Figure 7. Among the quantified alcohols, glycerol showed minimal variation during fermentation. Its concentration remained relatively stable across treatments, with values ranging from 0.12 mg/g at time zero to 0.18 mg/g in E1-East and E1-Central after 36 h. This consistency suggests that glycerol synthesis occurred at low but detectable levels, primarily through yeast metabolism during sugar conversion, as also observed in other spontaneous fermentations.

In contrast, ethanol concentrations increased significantly across all treatments, reflecting the expected outcome of alcoholic fermentation by yeasts. The most pronounced increase was observed in E3-C, where ethanol reached 1.24 mg/g. The higher ethanol production in E3 may be attributed to enhanced yeast metabolism under warmer and semi-anaerobic conditions, which favor the activity of *Saccharomyces cerevisiae.*

The presence of ethanol only at later fermentation stages suggests that initial aerobic conditions limited its production early on. Its accumulation after 36 h also coincides with increasing thermal retention and oxygen restriction—factors that promote fermentative metabolism. These results confirm the establishment of microenvironments that support active yeast fermentation, particularly in treatments with greater insulation or sun exposure.

### 3.5. Sensory Analysis

**Figure 8:**
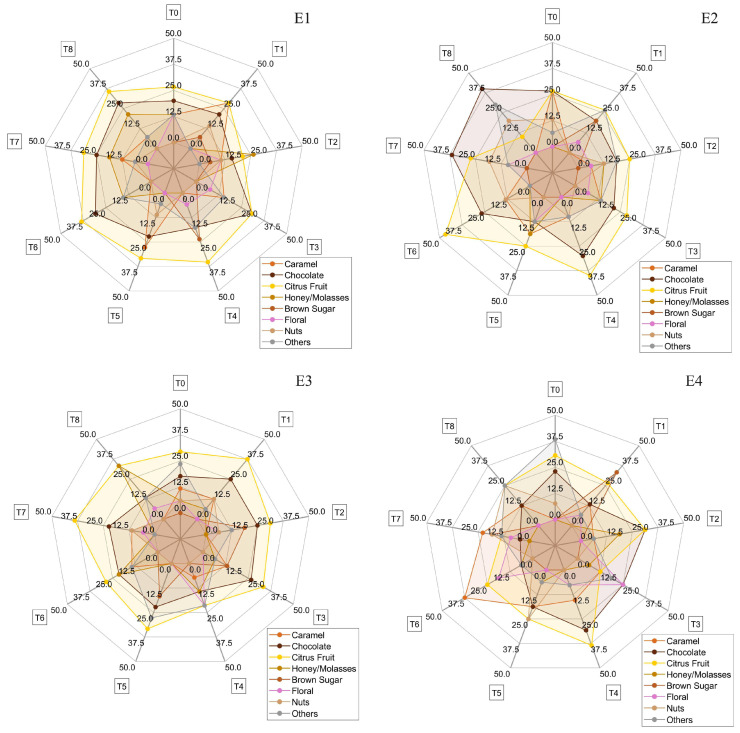
Sensory analysis of coffee for the different fermentation processes. E1: asphalt patio, E2: on pallets E3: in steel containers under the sun, E4: in steel containers in the shade.

Sensory notes play a vital role in determining the quality and value of coffee, directly reflecting consumer market preferences and the effectiveness of fermentation methods. A detailed analysis of sensory attributes provides valuable insights for producers, researchers, and the industry as a whole, promoting agricultural practices and processing techniques that elevate coffee quality to higher levels. Figure 8 displays the sensory descriptors identified in the cupping tests for each treatment (E1 to E4), with radar plots indicating the relative intensity or frequency of each descriptor mentioned. All coffees evaluated scored above 80 points, qualifying as specialty coffees. While no statistically significant differences were observed among the treatments (*p* > 0.05), treatment E2 showed a slightly higher intensity for descriptors such as citrus fruits, honey/molasses, and caramel.

Sensory attributes such as floral, nuts, and brown sugar also appeared frequently among the treatments. Although the other treatments did not show differences in sensory scores, they demonstrated different sensory complexities, both in descriptors and their intensities, reflecting the diversity of fermentation methods used. These variations highlight the influence of the specific conditions of each method on the final sensory profile of the coffee, resulting in unique flavor and aroma experiences for each batch.

In experiment E1, 15 sensory descriptors were identified. Chocolate was present in all treatments, being most prominent in the descriptions of evaluators in the treatment positions T3 and T6 (both 30.77%). Citrus fruits were also present in the descriptions of all treatment positions, especially in T4, T6, and T8 (35.29%, 38.46%, and 35.71%, respectively). Honey/molasses appeared significantly in the descriptions of positions T2 and T8 (26.32% and 21.44%, respectively), but was absent in T1, T3, T4, and T5. Caramel was described uniformly, with the highest percentage in T1 (28.57%), except in T4 and T6. Other descriptors, such as floral, brown sugar, almonds/nuts, peach, and pepper, were found in specific treatments. Heat retention favored the formation of complex sensory compounds, presenting high diversity and intensity of sensory attributes, with a predominance of chocolate, citrus fruits, honey/molasses, and caramel.

In experiment E2, using the volcano on pallets, 23 descriptors were identified by the Q-graders. Chocolate was predominant in all positions of the experiments, with the highest mention in T6 (46.67%). Citrus fruits were elevated in all positions, especially in T4 (40.00%). Caramel was present in all positions, except for T1, T7, and T8. Brown sugar was notable only in T1 (20.00%), while roasted peanuts appeared only in T7 but with high intensity (41.67%). Floral, pepper, and sweet had sparse and less intense distribution. Other descriptors such as almonds/nuts, spices, red fruits, and yellow fruits were also observed. Heat loss and ventilation may have influenced the formation of some sensory compounds, resulting in greater sensory diversity but with a reduction in the intensity of complex sensory compounds such as honey/molasses.

In experiment E3, using a volcano in a container under the sun, 22 descriptors were reported. Chocolate was present in all positions, with a high number of mentions in T1, T2, and T3 (25.00%, 25.00%, and 26.67%, respectively). Citrus fruits were elevated in all positions, especially in T1 and T7 (31.25% and 38.89%). Caramel had fewer mentions than in E2 and did not appear in positions T2, T5, and T8. Honey/molasses was significantly mentioned in T8 (33.33%), while yellow fruits were cited only in T2 (6.25%). Brown sugar, floral, and nuts had varied distribution, with a notable presence in specific positions. The diversity of sensory attributes indicates efficient formation of complex compounds, favored by high temperature and solar exposure. The central position T0 showed high sensory complexity with nine descriptors, including red and dried fruits, sweet, floral, and nuts. There was good formation of complex sensory compounds, similar to E1, with a predominance of chocolate, citrus fruits, caramel, and brown sugar.

In experiment E4, using a volcano in a container in the shade, 20 descriptors were identified. Chocolate was predominant in all positions, with a high number of mentions in positions T2 and T4 (31.25% and 30.77%). Similarly, citrus fruits were also reported in all positions, especially in T2 and T4 (31.25% and 38.47%). Caramel was uniformly distributed, except in T0, and had the highest number in T6 (37.50%). Brown sugar had a high number of mentions in T1 (33.33%), while yellow fruits were cited 23.08% in T0. Floral, nuts, honey/molasses, and tropical fruits had varied distribution in specific positions. Tropical fruits appeared only in experiments using containers E3 and E4, indicating the influence of the treatment type.

## 4. Discussion

This study evaluates the impact of volcanic fermentation on the physicochemical and sensory properties of Arabica coffee, contributing new insights into this emerging post-harvest method. This technique involves forming conical piles of coffee called “volcanoes” to provide specific conditions for biotransformation’s, which can be considered an innovative technique in post-harvest coffee processing. Like other fermentation methods, volcanic fermentation promotes exothermic reaction where energy is released for biochemical reactions [21]. Temperature plays an important role as it can directly influence the microbiota involved in the process, the starter cultures, and the degree of colonization on the coffee, consequently interfering with the rate of mucilage degradation and the fermentation time [22,23,24].

Since the microbiota is responsible for transforming the mucilage into volatile compounds during fermentation, a temperature variation, as observed in this study (16.6 to 44.1 °C inside the bioreactor, 16 °C to 22 °C ambient temperature), may have significantly influenced the profile of volatile compounds formed and the acidity of the coffee produced during fermentation [24,25]. This temperature behavior is consistent with previous studies that highlight the role of temperature in accelerating enzymatic and microbial reactions during coffee fermentation [21,25].

The fermentation environment, including the material in contact with the coffee mass, also impacts thermal dynamics. Surfaces like asphalt can retain heat and enhance thermal exchange, promoting microbial activity [25,26]. However, heterogeneous surface temperatures may result in uneven fermentation. The use of pallets facilitated airflow and improved thermal dissipation, potentially favoring more controlled fermentation. While low microbial activity can extend fermentation time, it may also enhance the complexity of aroma and flavor compounds [24,27].

Regarding the direct exposure of coffee to the sun, besides increasing the fermentation rate, it can cause overheating and consequently the production of undesirable compounds [24,28]. In contrast, fermentation under shaded conditions may allow for a slower and controlled evolution of volatile and acid compound production, resulting in coffee with a better sensory balance [24]. Thus, thermal efficiency can result in different fermentations, impacting the final sensory complexity of the beverage.

Variations in temperature and fermentation time can influence the formation of chemical compounds that affect both the color and physical appearance of coffee beans [11,13,29]. In the present study, all volcanic fermentation treatments showed a similar color profile in roasted beans (*p* > 0.05), with ΔE values ranging from 5.6 to 8.4. Although color is recognized as a key indicator of food quality and strongly influences consumer acceptance in the coffee market, especially regarding visual appeal and commercialization, no significant differences were observed between treatments. This uniformity is consistent with the findings of Rodriguez et al. [30], who reported that although different processing methods alter the color of green beans, such differences tend to disappear after roasting due to pigment homogenization from thermal reactions such as caramelization and Maillard browning. Similar observations were noted by Lee et al. [31], confirming that roasting reduces color variation caused by prior fermentation.

However, the texture of fermented coffee beans can be influenced by various factors, including the processing method, fermentation duration, bean quality, and other post-harvest management aspects, which directly impact bean quality. In this study, the instrumental analysis showed no significant changes in compression force, suggesting that the fermentation conditions did not compromise bean integrity. These findings are consistent with those reported by Rocha [7] and Silva [32], who also observed stable texture parameters in fermented Arabica beans under controlled fermentation protocols. Maintaining bean integrity is essential to minimize losses during drying, grinding, and roasting stages [32]

Throughout the fermentation process, the levels of sucrose, glucose, and fructose decreased in all treatments, indicating their active use as carbon sources by the fermentative microbiota. This behavior is expected in Cerrado-grown coffee cherries, which are naturally rich in sugars and thus support microbial metabolism during spontaneous fermentations [7,12,32]. Although reductions were observed for all three sugars, the fermentation time was not sufficient to fully deplete the sugar content. Notably, fructose remained at relatively high levels, a common outcome in coffee fermentations, since reducing sugars such as glucose and fructose are more readily metabolized than non-reducing sugars like sucrose [12,33,34].

In parallel, citric, malic, succinic, lactic, and acetic acids were detected both at the beginning and end of fermentation. The progressive increase in lactic and acetic acid, particularly in heat-retaining treatments, reflects the metabolic activity of lactic acid bacteria and acetic acid bacteria. The absence of propionic and butyric acids—typically associated with spoilage organisms such as *Clostridium* and *Propionibacterium* spp.—indicates that the fermentation conditions were well controlled, preventing undesirable microbial development.

It is emphasized that sucrose in coffee fruits can also decrease due to the conversion of sugar into glucose and fructose by the endogenous activity of invertase [34]. Surprisingly, the levels of glucose and fructose did not show a corresponding increase, but rather decreased due to their consumption during fermentation. Thus, the variation in the soluble solid content (°Brix) during coffee fermentation is an important indicator of the fermentation process intensity [35], where its reduction is related to the degradation of sugars and other soluble compounds during fermentation, directly influencing the coffee’s flavor and aroma profile [11,35,36]. It is worth noting that the variation in coffee soluble solid content is a direct consequence of the heterogeneous ripening of cherry coffees, which can result in different soluble solid content values.

During fermentation, the main sugars present in coffee—sucrose, glucose, and fructose—followed typical patterns of microbial consumption. Sucrose, although initially abundant, was partially hydrolyzed by enzymes such as invertase into glucose and fructose. However, instead of an increase in these monosaccharides, a gradual decrease was observed, particularly for glucose, indicating its rapid metabolism by yeasts and lactic acid bacteria as a primary carbon source.

Glucose was consumed more intensely than fructose, a trend well documented in the literature and attributed to the higher affinity of yeasts for glucose [37]. This explains the patterns seen in Figure 6, with a steady decline in glucose and, to a lesser extent, fructose. Sucrose remained more stable throughout the process, likely due to limited enzymatic activity or restricted accessibility within the fruit matrix.

Regarding organic acids (Figure 7), lactic acid concentrations increased significantly during fermentation, especially in treatments with higher thermal retention (E1 and E3), reflecting the activity of lactic acid bacteria. Acetic acid also showed a progressive increase, particularly in the final stages of fermentation, associated with the activity of *Acetobacter* spp. under microaerobic conditions.

Conversely, citric and malic acids—naturally present in coffee fruits—decreased over time due to their microbial degradation. These compounds serve as additional energy sources for yeasts and LAB, particularly in heterolactic fermentations, as previously reported by Bressani et al. (2020) [4]. This behavior reflects a dynamic balance between microbial production and the consumption of organic acids throughout the fermentation process.

During the first hours of fermentation, due to the consumption of these sugars by microorganisms, especially bacteria and yeasts, microbial counts increase along with the production of organic acids, alcohols, and other metabolic compounds, leading to mucilage degradation and pH decrease [25]. Thus, during fermentation, a decrease in soluble solid concentration and pH is observed, attributed to medium acidification due to microbial and enzymatic activity effects, along with the nature of products or metabolites generated over time [11,33,35,38]. In this study, the different volcano configurations and heat distribution did not significantly affect the final pH and soluble solid values between experiments after 36 h of fermentation.

The final pH values observed in this study were slightly lower than those typically reported for spontaneous coffee fermentations, which usually stabilize between 4.0 and 5.0 [39]. Previous studies have shown that elevated temperatures, especially under semi-anaerobic conditions, stimulate the activity of lactic acid bacteria and yeasts, leading to greater acid accumulation in a shorter time [25,37]. The consistent decrease in °Brix values indicates active microbial metabolism, as reported in other studies of spontaneous or semi-controlled coffee fermentations [25,40].

Besides sugars, coffee fruits naturally contain organic acids, such as citric, malic, and succinic acids, which influence the perceived acidity of the beverage [4] and the formation of volatile compounds during roasting, such as pyrazines, furans, and esters [6]. However, during coffee fermentation, acids are degraded, and their concentration increases due to microbial metabolism [41]. The concentrations of organic acids are modified by the degree of fruit ripeness, climate, variety, and processing type [8,42].

During fruit ripening, accumulated citric acid in the pericarp migrates to the seed, while high concentrations of malic and oxalic acids are detected in the pericarps [43,44]. Additionally, heterofermentative lactic acid bacteria can reduce citric acid to lactic acid via the oxaloacetate pathway, contributing to a low citric acid concentration [17,43,44,45]. Yeasts can produce succinic acid through isocitrate oxidation via the glyoxylate cycle and reductive citric acid cycle [46], besides its production by *Bacillus* spp. and heterofermentative *Lactobacillus* [47]. Acetic acid is also a key component in coffee fermentation, synthesized mainly by bacteria of the genus *Acetobacter* [48,49] and some species of *Enterobacter* spp. and *Lactobacillus* via the heterolactic pathway [10]. Positive accumulation of acetic acid can also correlate with warmer ambient temperatures [50], as in the experiment (maximum of 22 °C). The observed increase in lactic acid and acetic acid agrees with findings from Bressani et al. (2020) [4], who demonstrated that spontaneous fermentations involving *Lactobacillus* and *Acetobacter* species produce similar acid profiles in specialty coffee.

Lactic acid is also one of the main organic acids produced during coffee fermentation. [6,51]. Its production is primarily associated with lactic acid bacteria (LAB) [17], although it can also be produced by a wide range of bacteria and fungi [47]. LAB is a heterogeneous group with great species diversity. Besides producing lactic acid, some species also produce ethanol, CO_2_, and acetic acid [52]. In complex fermentation processes, this microbiota can ferment citric acid as a carbon source [53] and decarboxylate malic acid, resulting in decreased citric and malic acid levels detected at the beginning of processing and increased lactic acid concentration [18,54].

Yeasts can also consume malic acid, converting it into ethanol through malo-ethanol desacidification [55]. However, this is not the main pathway for ethanol production. The primary pathway is alcoholic fermentation, mainly synthesized by *Saccharomyces* sp., an important microorganism found during spontaneous fermentation in the Cerrado Mineiro region. Glycerol, on the other hand, is mainly produced by sugar metabolism by yeasts [16]. The increase in ethanol in E3 and E4 is in accordance with studies by Silva et al. (2013) [1], who showed that heat-retaining fermentations stimulate yeast metabolism, particularly *Saccharomyces cerevisiae*, leading to higher ethanol and glycerol production [56].

Although ethanol was only detected in selected treatments (notably E3 and E4), its absence in other samples may be attributed to suboptimal fermentation conditions for yeast activity or to ethanol concentrations falling below the detection limit of the analytical method. In the early stages of fermentation (up to 24 h), limited yeast activity—possibly due to higher oxygen availability and lower temperatures—may have restricted ethanol production. Its presence only after 36 h in specific treatments reflects a shift to more anaerobic and thermally favorable conditions. This variation is consistent with the sensitivity of *Saccharomyces cerevisiae* to environmental factors such as temperature, oxygen availability, and substrate composition.

As for propionic and butyric acids, their absence throughout the process is actually desirable as these compounds are typically produced by spoilage microorganisms such as *Clostridium* and *Propionibacterium* spp., which develop under poorly controlled anaerobic fermentations. Their non-detection indicates that the volcanic fermentation protocol provided a clean and well-managed microbial environment [42,57].

This acidification process, with the production of organic acids during coffee fermentation, plays a promising role in creating specialty coffees with desirable sensory characteristics [4,18,43]. These compounds influence perceived acidity in coffee, an essential quality attribute combined with sweetness, bitterness, and aroma [15], affecting the beverage’s final sensory profile [56].

Citric acid can impart acidic, fruity flavors, like citrus fruits and red fruits, and is appreciated during tasting [58]. Higher final concentrations of lactic acid may be related to a greater sensory perception of nuts, cocoa, and sweetness in coffee [25,37]. Low levels of acetic acid are desirable in coffee beverages and may be related to a sweet taste. However, an undesirable vinegary taste can be found in beverages from over-fermented coffee [59]. Both glycerol and ethanol are valued in various beverages; glycerol has a sweet taste and contributes to the beverage’s body, providing a smooth mouthfeel [16], while ethanol enhances the palate’s fullness in wine [60].

Citrus fruit, chocolate, and caramel notes were perceived in all treatments using the volcanic fermentation method. Additionally, other compounds related to sugar caramelization generated characteristic coffee flavors and aromas [43]. Sensory attributes like honey/molasses, floral, nuts, and brown sugar also appeared frequently among the treatments. The predominant descriptors—chocolate, citrus, and caramel—are consistent with those reported in other works involving Arabica coffees from the Cerrado Mineiro region [7,12,32]

Therefore, identifying the optimal fermentation conditions for producing specific alcohols and acids is essential in specialty coffee production. By understanding how fermentation conditions influence sensory notes, producers can optimize their processes to maximize their coffees’ quality.

## 5. Conclusions

The volcanic fermentation method proved to be effective in producing high-quality specialty coffees, regardless of sun exposure. Although solar conditions influenced the chemical profiles of the beans—leading to distinct sensory attributes—the fermentation process itself remained efficient and consistent, as indicated by the stability in pH and soluble solids across treatments. This suggests that the native microbiota adapted well to the different environmental conditions.

The choice of fermentation setup plays a critical role in shaping the thermal environment, which, in turn, influences the biochemical reactions essential for coffee flavor development. This study emphasizes the importance of selecting appropriate configurations and maintaining controlled fermentation conditions—such as temperature and ventilation—to enhance the chemical complexity and sensory appeal of the final product.

While the results are promising, further research is required to deepen our understanding of microbial dynamics and environmental factors in volcanic fermentation. Future studies should explore strategies to optimize the process, aiming to improve flavor consistency and maximize the potential of this innovative post-harvest technique in specialty coffee production.

## Data Availability

The original contributions presented in this study are included in the article/Supplementary Material. Further inquiries can be directed to the corresponding author.

## Supplementary Materials

The following supporting information can be downloaded at: https://www.mdpi.com/article/10.3390/foods14081368/s1, Figure S1. Representative images of the volcanic biotransformation process. Volcano setup on patio (E1); pallet structure (E2); inclined steel container under the sun (E3); inclined steel container in the shade (E4). Photos taken during fermentation in Patos de Minas, Brazil. Note: Containers used in experiments E3 and E4 were inclined at 45° to simulate the slope of a volcanic surface and to promote internal heat circulation.

## Appendix A

**Table A1:** Treatments performed during volcanic coffee fermentation.

Experiment	Process Type: Volcano	Coffee Type	Time (Hours)	Coffee Quantity (Liters)
E1	Asphalt patio	Paraíso/Cherry	36	4000
E2	Pallet	Paraíso/Cherry	36	4000
E3	Containers under the sun	Paraíso/Cherry	36	4000
E4	Containers in the shade	Paraíso/Cherry	36	4000
