# Supplementary material for: Coffee Biotransformation in Volcanic Process: A Chemical and Sensory Analysis
Source: Foods. 2025 Apr 16;14(8):1368. doi: 10.3390/foods14081368 (PMC12027445; doi:10.3390/foods14081368)
Supplement: Supplementary file 1 [file foods-14-01368-s001.zip › foods-3568510-supplementary.pdf]

Supplementary Material

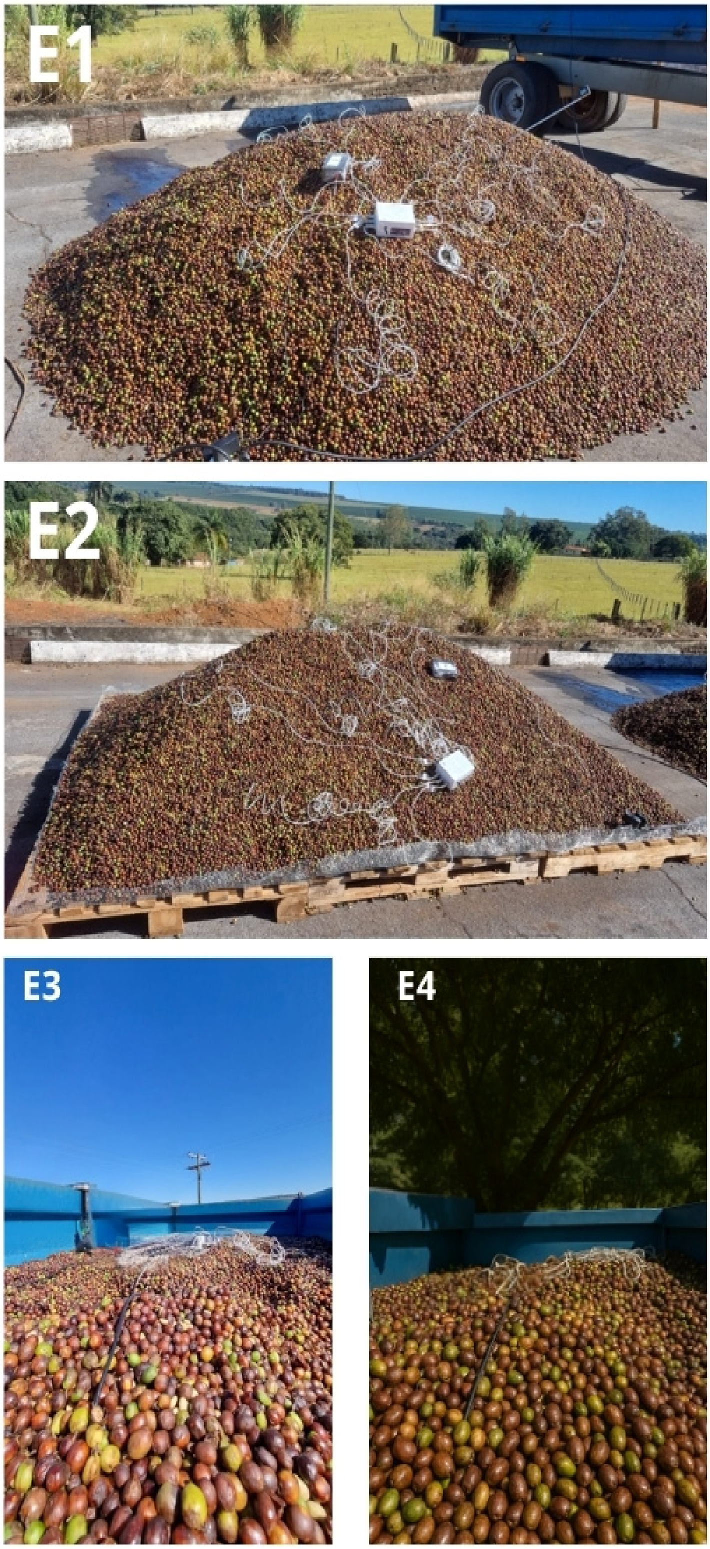

Figure S1. Representative images of the volcanic biotransformation process. Volcano setup on patio (E1); pallet structure (E2); inclined steel container under the sun (E3); inclined steel container in the shade (E4). Photos taken during fermentation in Patos de Minas, Brazil. Note: Containers used in experiments E3 and E4 were inclined at 45° to simulate the slope of a volcanic surface and to promote internal heat circulation.
